# Human Alveolar Echinococcosis after Fox Population Increase, Switzerland

**DOI:** 10.3201/eid1306.061074

**Published:** 2007-06

**Authors:** Alexander Schweiger, Rudolf W. Ammann, Daniel Candinas, Pierre-Alain Clavien, Johannes Eckert, Bruno Gottstein, Nerman Halkic, Beat Muellhaupt, Bettina Mareike Prinz, Juerg Reichen, Philip E. Tarr, Paul R. Torgerson, Peter Deplazes

**Affiliations:** * University of Zurich, Zurich, Switzerland; † University of Bern, Bern, Switzerland; ‡ University of Lausanne, Lausanne, Switzerland; 1 This author submitted this article to the University of Zurich as part of his Doctor of Medicine postgraduate degree requirement.

**Keywords:** Alveolar echinococcosis, *Echinococcus multilocularis*, epidemiology, fox (*Vulpes vulpes*), zoonosis, incidence, Switzerland, research

## Abstract

We analyzed databases spanning 50 years, which included retrospective alveolar echinococcosis (AE) case-finding studies and databases of the 3 major centers for treatment of AE in Switzerland. A total of 494 cases were recorded. Annual incidence of AE per 100,000 population increased from 0.12– 0.15 during 1956–1992 and a mean of 0.10 during 1993–2000 to a mean of 0.26 during 2001–2005. Because the clinical stage of the disease did not change between observation periods, this increase cannot be explained by improved diagnosis. Swiss hunting statistics suggested that the fox population increased 4-fold from 1980 through 1995 and has persisted at these higher levels. Because the period between infection and development of clinical disease is long, the increase in the fox population and high *Echinococcus multilocularis* prevalence rates in foxes in rural and urban areas may have resulted in an emerging epidemic of AE 10–15 years later.

## Abstract 2

An increase in fox population has led to an increase in incidence of human alveolar echinococcosis.

Human alveolar echinococcosis (AE), a hepatic disorder that resembles liver cancer, is a highly aggressive and lethal zoonotic infection caused by the larval stage of the fox tapeworm, *Echinococcus multilocularis* (*1*). The parasite is widely distributed in the Northern Hemisphere; the disease-endemic area stretches from North America through Europe to central and east Asia, including northern parts of Japan (*1**–**3*). Parasite eggs are shed into the environment in the feces of canid definitive hosts that harbor the adult parasite in their intestines. In addition, some of these eggs contaminate the fur of infected definitive hosts. Humans and intermediate host animals acquire the infection by ingesting *E. multilocularis* eggs in contaminated food or water or by having close physical contact with infected foxes, dogs, or host feces. In Europe, *E. multilocularis* exists predominantly in a cycle of wild animals that includes red foxes (*Vulpes vulpes)* as main definitive hosts and several vole species as intermediate hosts. In the United States and Canada, the coyote (*Canis latrans*) has also been shown to be a suitable host for this parasite; in Arctic regions, the artic fox (*Alopex lagopus*) is the principal definitive host (*1*). Domestic dogs are also highly susceptible definitive hosts (*4*); in some areas, such as Alaska (*5*), People’s Republic of China (*6*), and Europe (*7*), they can play an additional or even the dominant role as an infection source for humans.

The documented area of *E. multilocularis* endemicity in Europe has recently increased (*3**,**7**,**8*). However, whether this increase results from a true extension of the geographic range or simply increased detection in populations of wildlife not previously investigated is still unclear (*1*). Similarly, new distribution ranges have been reported in North America. In addition to being found in the tundra zone of Alaska and Canada, the parasite has now been recorded in 3 Canadian provinces and an additional 11 contiguous US states (*9*).

Important changes have occurred in the population dynamics of foxes in central Europe. Between 1970 and the mid-1980s, fox populations decreased during an epidemic of rabies. After the successful establishment of anti-rabies vaccination programs, fox populations increased almost 4-fold (*10*). At the same time, fox habitat extended into urban areas and is still progressing. For example, large fox populations have now become established in all major cities and towns in Switzerland. In the largest city, Zurich, the number of foxes shot or found dead within the city boundaries increased 20-fold since 1985 (*10*). The reasons for the increase in the fox populations have been attributed to ecologic factors, the successful vaccination campaign against fox rabies, and the increase in anthropogenic food supplies (*7*,*10*).

In the core European area for AE, which includes Switzerland as well as neighboring France and Germany, high prevalence rates (35%–65%) of *E. multilocularis* in foxes have been consistently recorded (*3**,**7**,**8**,**11*). However, several studies have shown that the prevalence rates have been increasing in certain regions. For example, recent parasite density estimates in southwestern Germany were 10× higher than estimates before 1990 (*3**,**8*), and unexpectedly high prevalence rates in several urban fox populations have been reported (*7*). The combination of increased fox populations and increased parasite prevalence within these populations has led to a considerable increase in the overall parasite biomass per surface unit.

A question of major public health importance in central Europe is whether the growing fox populations that have high prevalence of infection and the colonization of densely populated urban areas by foxes could increase risk for transmission of AE to humans and lead to an increase of clinical cases. Until 2000, no statistically significant increase in AE had been recorded in Switzerland (*1*). We report countrywide annual incidence rates of human AE in Switzerland in recent years (1993–2005) and compare them with incidence data for previous years (1956–1992). The European Echinococcosis Registry (*11*) gives summary data for 559 cases of AE from central Europe between 1988 and 2000, which includes data from 112 of the Swiss cases in the present report. The incidence data were related to the fox population dynamics. Because Switzerland has been consistently collecting comprehensive data on AE in humans for 50 years, conditions for such long-term assessments are favorable.

## Methods

We retrieved retrospective data of extensive AE case-finding studies covering all of Switzerland for 1956–1992 (Table). We included additional data from the Swiss National Center for Echinococcosis, collected when echinococcosis was a reportable disease (1987–1996), and data for 1996–2005, collected from databases of the University Hospitals of Zurich, Berne, and Lausanne, the 3 major centers for AE treatment in Switzerland.

**Table:** Data from reported case-finding studies on human alveolar echinococcosis, 1956–2005, Switzerland

Study (reference no.)	Years	No. cases	Mean annual incidence/100,000 population	Mean age ±SD, y	Sex, no. (%)	
					Male	Female
Drolshammer (*12*)	1956–1969	122	0.15	54.2 ± 18.2	65 (53)*	57 (47)
Gloor (*13*)	1970–1983	145	0.16	55.0 ± 16.0	79 (54)*	66 (46)
Eckert (*14*)	1984–1992	71†	0.12	52.0 ± 17.7	33 (46)	38 (54)
This study	1993–2000	60	0.10	52.5 ± 18.4	26 (43)	34 (57)
This study	2001–2005	96	0.26‡	54.5 ± 17.3	42 (44)	54 (56)
Total	1956–2005	494	0.15	54.0 ± 17.3	245 (49.7)	248 (50.3)

*Proportion of cases in male patients was significantly higher during 1956–1983 than 1984–2005 (p<0.05). †Six more cases from 1984–1992 have been included in this study. ‡Significantly increased compared with1984–2000 (p for trend <0.01).

Furthermore, we analyzed serodiagnostic data compiled from the 3 main diagnostic laboratories for parasitic diseases in Switzerland that offer reliable (and methodically comparable) immunodiagnosis of human AE (Institutes of Parasitology, Universities of Zurich and Bern, Swiss Tropical Institute in Basel). Cases that were diagnosed primarily on the basis of serologic testing results were further assessed by sending questionnaires to involved family doctors or by conducting retrospective analysis of the patients’ history obtained from the treating hospitals.

Inclusion criteria were as applied previously (*11*). These were 1) diagnosis of AE by positive, species-specific serologic testing (*15*), 2) AE-characteristic imaging findings, and 3) if available, AE-characteristic histopathologic findings and species-specific molecular analysis by PCR. Seropositive persons who lacked characteristic imaging features or histologic or molecular diagnosis were excluded from the study. All data were entered by using only personal initials, birth date, and sex; patients remained anonymous. Data were collected in a single database (Microsoft Excel; Microsoft Corp., Redmond, WA, USA) to prevent multiple counting of patients.

For cases that occurred during 1993–2005, for which data on the clinical records were available, we used the PNM (primary, neighboring, metastasis) staging system, specifically, location and extension of the primary lesion, involvement of neighboring organs, and presence or absence of metastasis (*16*). We determined the stage of disease (stages I–IV) by using this PNM system. Stages I and II are more likely to represent subclinical disease and are often diagnosed by chance. Such cases, if detected early, are more amenable to curative resection. Patients with PNM stage III or IV are more likely to have advanced clinical disease. They have more limited treatment options, such as individualized interventional measures and lifelong chemotherapy (*17*). The stage of cases diagnosed during 1993–2000 was compared with stage of cases diagnosed during 2001–2005 by converting the stage to a score. For example, patients with stage I disease were scored as 1 and those with stage IIIb disease were scored as 3.5. To determine whether earlier or later diagnosis during different periods might partly account for any trend in the change of incidence, the mean score of cases diagnosed during 1993–2000 was compared with mean score of those diagnosed during 2001–2005. PNM scores were available for 50% (51 cases) of the 2001–2005 cases and for 40 (68%) of the 1993–2000 cases. For 2001–2005, the average age of case-patients with available PNM scores was 54 years (SD 17 years). This average did not significantly differ from the average of 50 years (SD 18 years) of case-patients with PNM scores for 1993–2000 (p = 0.19, Student *t* test). Likewise, the proportion of male patients with scores for 2001–2005 (37%) did not significantly differ from the proportion of male patients with scores for 1993–2000 (44%) (p = 0.35, Fisher exact test). Therefore, we found no evidence of bias due to incomplete data.

We retrieved data regarding fox population sizes from the Swiss federal hunting statistics (www.wild.unizh.ch/jagdst). These data, based on annual hunting numbers, indicate trends over a long period of time (*18*). Human population data for Switzerland originated from the Swiss Federal Statistical Office (www.bfs.admin.ch).

To perform our statistical calculations, we used Microsoft Excel. Differences in human incidence rates and differences in numbers of reported foxes were analyzed by χ^2^ test for trend. Proportions of male and female patients and the proportion of patients who had radical liver resection were analyzed by χ^2^ test. Data for PNM scores were tested for normality and compared by using the Student *t* test. To smooth out annual fluctuations and better visualize longer term trends, we present data for number of foxes and incidence of human AE cases as 5-year moving averages.

## Results

A total of 494 cases of human AE were diagnosed in Switzerland during 1956–2005. The mean age at time of diagnosis (54 years, range 12–89) did not change significantly over time (Table). The numbers of male and female patients were similar, although the trend was toward a decreasing proportion of male patients over the observation period. Male patients accounted for 53%–54% of patients during 1956–1983 compared with 43%–46% during 1984–2005 (p<0.05) (Table). The incidence of human AE during the observation period is shown in the Figure. The highest annual incidence per 100,000 was recorded in 2003 (0.38; 28 new cases); the lowest, in 1996 (0.04; 3 new cases). The mean annual incidence per 100,000 was 0.10 during 1993–2000 and increased to 0.26 during 2001–2005 (p<0.01) (Figure, Table). The estimated fox population increased ≈4-fold during 1984–1993 (Figure) (p<0.001).

**Figure:**
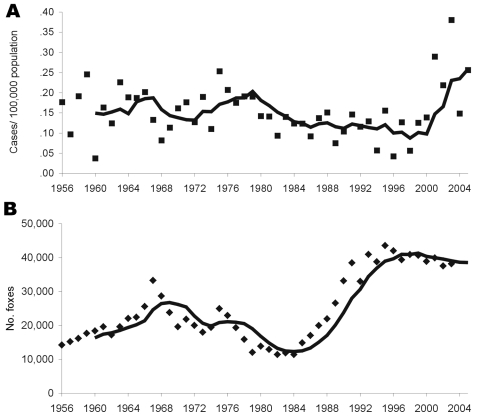
Actual data points with moving 5-year average for annual incidence of human alveolar echinococcosis in Switzerland (A) and annual number of foxes hunted per year in Switzerland (B), used as a fox population density marker.

The mean PNM score for cases diagnosed during 1993–2000 was 2.41 (SD 1.3), which was not significantly different from the mean PNM score of 2.44 (SD1.2) for cases diagnosed during 2001–2005. The proportion of patients who had a radical liver resection was highest during 1996–2000 (75.3%) and lowest during 1991–1995 (37.5%) and 2001–2005 (54.9%) (p<0.001).

## Discussion

The incidence of human AE in Switzerland has exhibited 2 trends during the past 50 years. After a slow but steady decline during 1956–1999, incidence has significantly increased since the year 2000 (Figure). A reasonable explanation for this finding may be the urbanization of the *E. multilocularis* cycle, which has resulted in an increase in the number and proportion of infected urban foxes in areas with high human populations, thereby increasing the infection risk for the human population (*7*). The increase of the fox population density started in ≈1985, some 10–15 years before the increased numbers of human AE cases (Figure). This temporal delay is consistent with the suggested latent or asymptomatic period of 5–15 years before development of clinically apparent AE in humans (*19*). Improved diagnostic accuracy, due to modern imaging technologies such as computerized tomography (*20*)*,* is unlikely to account for this increase. Such improvements in diagnostic accuracy would be expected to lead to earlier detection with a shift to PNM stages I and II at the time of diagnosis, but such a trend has not been recorded. In addition, earlier, more local investigations have already indicated an increase in AE seropositivity in defined human populations at risk in central Europe (*21**,**22*) without a concomitant increase in clinical AE cases. An increase in the rate of AE seropositivity that precedes that of clinical AE is therefore consistent with increased exposure and a temporally delayed rise in the number of clinical cases.

The use of foxes recorded in hunting statistics or hunting indices as a measure of the fox population is susceptible to bias and should be used only to describe trends from large areas (>1,000 km^2^) over long periods (>5 years) (*18*). For this reason, the temporal trends in the size of the fox population were estimated by using hunting returns compiled for 50 years for the entire territory of Switzerland (42,000 km^2^). Therefore, we can conclude that the incidence of human AE appears to be increasing in Switzerland and that this increase was preceded 10 years earlier by a parallel increase and urbanization of the fox population.

The potential extent of this emerging epidemic of human AE cannot be predicted. Future trends will depend on the intensity of present and future contamination of the environment with *E. multilocularis* eggs as well as on the number of susceptible persons exposed to the parasite. In this respect, the human AE epidemic appears analogous to that of human variant Creutzfeldt-Jakob disease, for which predictions of future disease trends have been hampered by uncertain knowledge of incubation periods and unknown relationships between the risk for disease and host factors (*23**,**24*). Nevertheless, the temporal proliferation of *E. multilocularis* biomass in the main definitive host has increased the infection pressure for a large part of the human population in Switzerland. Likewise, other susceptible canid species involved in the life cycle of the parasite could present additional threats, in Europe and elsewhere. Within the past decade, for example, coyotes in the United States have become established in suburban areas with moderate to dense human populations (*25*). Because this species is a suitable definitive host of *E. multilocularis (9)*, risk for transmission to humans in the United States and Canada may increase markedly.

In conclusion, public health authorities in echinococcosis-endemic areas should establish coordinated systems of continuous surveillance and risk assessment, combined with measures to reduce illness and death from AE in human populations (*1*). Furthermore, new control strategies, including strategic deworming of foxes and other wild canids by using anthelminthic baiting options, should be further evaluated and developed. Such strategies should preferably target suburban areas that have high human and wild canid population densities (*7*).
